# Annotations

**Published:** 1906-03-17

**Authors:** 


### ANNOTATIONS.

### How Typhoid Fever is Cultivated.

A striking example of official apathy towards gross sanitary defects is furnished by the report to the Local Government Board of Dr. Spencer Low upon enteric fever at Sutton Bonnington, in the Leake rural district of Nottinghamshire. Dr. Low's examination of the drinking water revealed a condition of things which, but for his precise language, might be looked upon as inconceivable. The two villages concerned, Sutton Bonnington and Normanton, are situate upon a flat area of land a few feet above the level of the river Soar. The superficial strata in this river valley are sand and gravel, and the villages derive their drinking water from shallow wells dug in these beds. Not only do several of the villages above Sutton Bonnington discharge their liquid refuse into the river, but nightsoil is brought into the district by rail and canal boat from Nottingham, where enteric fever is not uncommon, and is spread upon the land in the valley. The common method of disposing of excrement in the district is by means of privy middens, the pit being merely an uncovered hole in the ground, so that, in effect, the privy pit and the well are situate in the same superficial strata. Parts of both villages drain directly into tortuous ditches, which have no fall, and contain stagnant sewage and foul-smelling sludge. The only approach to a sewerage system was constructed some years ago, and consists of stoneware pipes laid along part of the main street of Sutton Bonnington. These pipes were jointed with clay when laid down, because, as Dr. Low was informed by the Inspector of Nuisances, the Council considered that sooner or later an efficient sewerage scheme would become necessary, and that when that time arrived the sanitary pipes, being only "gently jointed" with clay, would be the more readily taken up, and would then be fit for use again. It is quite clear that conditions favourable to the diffusion of fever are not only encouraged at Sutton Bonnington, but are absolutely created there; and that the humbler class of residents are improperly deprived of the protection which it has been the purpose and intention of the law to provide for them.

### The Poor-law as a Contagious Disease.

At the Stepney Distress Committee a letter was recently received from the Central Distress Committee for London, suggesting that all the men sent to the colony at Hollesley Bay should be over forty-five years of age, and that before being sent there they should be examined by the district medical officers to see if they were physically fit for the life of the colony. On the face of it there seems to be nothing offensive in the suggestion, yet strong objection was taken to it on the ground that the medical examination brought the men into close contact with the Poor-law. One would think that the Poor-law was a contagious disease. The men who go to Hollesley Bay are quite willing to accept free maintenance from the hands of the charitable, and for convenience the committee who administer this charity are selected in part from the guardians of the poor, because these know something of the conditions of poverty in the district. The Guardians also know something of the medical men whom they have appointed to attend the poor in their union, and it is reasonable, therefore, that the district medical officers should be regarded as the right men to see and report upon the health of any men sent to a labour colony. As a matter of fact, anyone who knows the kind of men who ask to be relieved as "unemployed" is aware that they and their families have in most cases already been attended by the parish doctor. Hardly one of the "unemployed" would refuse out-door relief if it were offered. The distinction between them and a pauper is simply that of being refused one kind of charity and being offered another, and it would puzzle any sane man to say how their character or independence would be affected by having their chests sounded by a Poor-law doctor. Yet the Stepney Committee demand that the examination be made by an outside medical man. For their protégés must not be brought into close contact with the Poor-law. Verily the notions of sentimental socialism are wonderful!

### A Medical Test for Motorists.

If there are motorists who think that the authorities in this country chastise them with whips, they will doubtless be of opinion that in France it is intended to scourge them with scorpions. According to some new regulations across the Channel, no one will in future be granted a license who is not able to prove, in addition to the possession of the necessary technical knowledge, exemption from any physical infirmity which would tend to involve unfitness for the control of a car. This means, of course, that a medical examination must be submitted to before a motorist can be licensed. The condition is objected to, but there is obviously a great deal to be said in its favour, both in the interests of the public and of the drivers themselves, whether paid chaffeurs or amateurs. It is not compatible with accepted ideas of security to either pedestrians or occupants of vehicles that persons whose eyesight is in the least impaired, whose hearing is not acute, whose hand is rendered unsteady by drinking habits, or who suffer from fits of nervousness, should be allowed to add enormously to the risks of serious accidents on the road. We are by no means sure that the time for observing similar precautions with respect to motoring in the United Kingdom is not approaching. It is a reasonable assumption that anyone who cannot pass the necessary medical examination is no more properly qualified to steer a motor-car than a colour-blind ship's captain to assume responsibility for the safety of his vessel or a deaf engine driver of his train.

